# Dentition

**Published:** 1898-06

**Authors:** 


### Dentition.

The views in regard to the effects of teething on the health of children have undergone a complete change. The doctors of the old school were accustomed to lay at the door of dentition most of the diseases occurring between the ages of six months and two years. At the present time many practitioners go to the other extreme, and deny that teething causes any symptoms whatever. Probably the truth lies in the happy medium, for while the importance of dentition in causing disease has doubtless in the past been greatly over-estimated, yet it is true that reflex symptoms, due to teething, may arise, often of a serious nature. In *L'Independence Medicale* of January 26th M. G. Ausset gives expression to some pertinent remarks on the matter. He says: "The accidents due to dentition are both local and general. * * * A dry, nervous, and sometimes croupy cough often accompanies dentition, and in the sickly and rachitic there may be spasms of the glottis and laryngismus stridulous. In some infants the appearance of each tooth is accompanied by a bronchial catarrh. None of these symptoms due to dentition have any special characteristic serving to distinguish them from symptoms otherwise caused, so that the diagnosis is often difficult. A few rules will, however, be of assistance. The first thing to be done is to ascertain the previous health of the child, for nine times out of ten if a healthy child, hygienically brought up, is suddenly affected by any of the above-mentioned accidents, we may affirm they are due to dentition. Reflexes, due to dentition, begin abruptly and disappear as suddenly as they came. They are not in themselves true diseases, but they may predispose to real affections. Dentition cannot create tuberculous meningitis, but in the case of a child predisposed by heredity and by previous localizations of tuberculosis, the congestion of the meninges, induced by dentition, might create a "locus minoris resistentiæ" wherein the bacilli of tuberculosis would find a suitable environment for its development. It may here be said that the custom prevailing with many practitioners of lancing the gums on the slightest provocation is one that in the majority of instances does more harm than good. There are occasions when lancing is necessary and affords great relief, but its indiscriminate practice is to be greatly deprecated.—*Pediatrics.*

